# Alphabet of Medical Botany, for the Use of Beginners

**Published:** 1834-04-01

**Authors:** 


*Alphabet of Medical Botany, for the Use of Beginners.* By JAMES RENNIE, M.A., Professor of Zoology, King's College, London.—London, 1834. 18mo. pp. 152.

*Alphabet of Botany, for the Use of Beginners.* By JAMES RENNIE, M.A., &c.—London, 1833. 18mo. pp. 192.

THE peccant abecedarian, the man of alphabets and errors, —Professor Rennie, in short,—has so extraordinary a fecundity of these pigmy treatises, that it would require a whole battalion of reviewers to keep pace with the innumerable litters of this literary rabbit; but it fortunately happens that a small number only of his productions fall within our province, and we leave to other censors to determine how much the professor, or his amanuenses, may know of Astronomy, Natural Theology, Perspective, Agriculture, Euclid's Geometry of Straight Lines, &c., on which, and many other subjects, he has Alphabets in "forward preparation."

In the commencement of the Alphabet of Medical Botany Mr. Rennie says, "I have followed the system of Linnæus, as the easiest for a beginner; but have taken care to point out, at the same time, the order to which each plant belongs in the system of Jussieu, as it has been improved by De Candolle, Richard, and others." (P. 2.) The latter part of our author's promise has hardly been fulfilled in a single instance: the deviations both from Jussieu and from his followers are constant; and we shall presently see that the Professor is very often inconsistent with himself. Whether a *Jussieuan* order, in the Alphabet before us, means one of the orders devised by Jussieu, or one of those introduced by his followers, is not quite so clear as might be wished; for the word is used in both senses, and sometimes in neither: thus, at page 13, the Zedoary, Ginger, and Cardamom, are said to belong to the Jussieuan order Scitamineæ. Now, there is no such order in the system of Jussieu, but it is one of the natural orders of Linnæus. The plants just mentioned belong to Jussieu's natural order Cannæ: this has been subdivided by Robert Brown, who reintroduces the Linnæan name for one of the sections: but of all this our compiler appears to be unaware; and although, at p. 13, he says the Zedoary, Ginger, and Cardamom, belong to the *Scitamineæ,* at p. 114, when describing the Jussieuan system, he says they belong to the order *Amomæ!* [Amomeæ?] At p. 15, the Gratiola officinalis is referred to the *Jussieuan* order *Personatæ.* Now, this order is not one of Jussieu's, but belongs to the natural system of Linnæus. Again, in the same page, the olive is said to belong to the Jussieuan order *Oleinæ,* while at page 119 it is referred to the *Jasmineæ.* Any one acquainted with the subject sees clearly how and why the professor was misled: the fact is, that the *Oleinæ* are one of the modern subdivisions of Jussieu's *Jasmineæ:* but how is the student to guess this?

There is no end of blunders of this kind. At pages 16 and 17, the Peppers are referred to the Jussieuan order *Urticeæ,* which belong to the 15th class of his system, and are the 98th order, being one of the diclinious Exogenæ or Dicotyledons; but at p. 112 they are referred to the order *Piperiteæ,* (Piperitæ, or Piperaceæ, we presume to be meant,) and here the Peppers are classed among the Monocotyledons.

That *Jussieuan* often does not mean the orders given by Jussieu himself, is evident even from the above examples; and that it often does not mean the modern modifications of the natural system, is equally evident. Had the improvements of De Candolle, Richard, and others, been really introduced, the author would have known that Cinchona, (p. 26 and 125,) Coffee, Ipecacuanha, &c., now belong to the natural order *Cinchonaceæ;* or, if he chooses to retain the term, *Rubiaceæ,* that the Madders (p. 23 and 125,) belong to the *Stellatæ.* At page 35, the Elm is said to belong to the Jussieuan order *Amentaceæ,* and at page 137 to the *Ulmaceæ.* The Flax-plant, at page 36, is placed among the Caryophylleæ, but at page 133 among the *Linaceæ,* a Rennieism for *Lineæ;* just as at page 39 we have *Colchiceæ* for Colchicaceæ, and *Acerideæ* for Acerineæ: the Jussieuan order is *Acera.* The Oxalis is referred to the *Geraniaceæ* at p. 56, and to the *Oxalideæ* at p. 130. At p. 60 the figure of the Pomegranate does not agree with the description in the text, as to its corolla. At p. 66 the description of the Aconite is altogether obsolete. Our abecedarian says it has no calyx: De Candolle says that it has a calyx, which is petaloid. The Alphabet affirms that it has a corolla formed of five petals; while De Candolle says the petals are two in number, &c. The accounts of Delphinium Staphisagria (p. 67), and of the Helleborus niger (p. 68), are equally faulty. The name Spartium scoparium (p. 83), is quite obsolete, the proper appellation being Cytisus scoparius.

Were we not tired of pointing out errors, we might give numerous instances of such ignorance as occurs at page 84, where the Lemon is called *Citrus medicus,* as if Citrus were masculine, and the specific name signified medical; whereas it ought to have been written Medica to agree with Citrus, and to indicate that the native place of the fruit is *Media.*

As to names, the Professor is very much behindhand. The Corsican worm-grass has long ceased to be called *Fucus Helminthocorton;* it is a species of Gigartina. The Lichen Islandicus is now called *Cetraria Islandica,* and the Scilla maritima, *Ornithogalum Squilla.* The errors in orthography are exceedingly numerous: thus, at p. 24, we have πεντα for πεντε; at p. 37, ἐξ for ἑξ; at p. 41, ἡπτα for ἑπτα; at p. 43, ὀκτο for ὀκτω; at p. 74, 'νημα for νημα; to say nothing of νημα being made a component part of *Didynamia* and *Tetradynamia.* At p. 115, we have Asarum longa for *longum;* and so on, for ever. Nor are we much pleased with our compiler for calling Asparagus *Sparrow grass,* and the Asparagineæ *Sparrow grasses,* as he does at p. 113. This should have been reserved for an Alphabet of Street-cries, with which we shall, no doubt, be favoured in due time.

This Alphabet of Medical Botany, passing over its errors of execution, is, even in its plan, a work of the most superficial kind. It consists of nothing more than what would constitute the heads of chapters, or the table of contents, of a really useful book. The Professor has copied his descriptions from well-known works; the mistakes only are his own.

For the sake of beginners, we must continue this painful dissection, and demonstrate a few of the errors with which the Professor has most plentifully besprinkled his Alphabet of Botany: we say a *few* only, as we should be ashamed to fill our pages with an exposure of all its faults. It errs both as to matter of fact and reasoning; the compiler, throughout his manifold blunders, keeping up the most unruffled self-complacency.

Mr. Rennie has heard something about a potato not being a true root; but that he does not know why, appears from his supposing a carrot to be in the same predicament.

"Upon the same principle which leads naturalists to rank the whale and the dolphin among land animals, and not among fishes, modern botanists do not consider carrots, potatoes, and the like, as roots, but as subterranean stems, because they perform the functions of stems rather than of roots." (P. 11.)

Were we to judge of the Professor's acquaintance with zoology from the above extract, we should suppose him to be a beginner even in the science which he professes; for, although the whale and the dolphin are classed among the *Mammalia,* they were surely never placed among *land animals.*

The Professor has no mercy on the modern theory of the modern metamorphosis of plants, but says,

"According to the partially fashionable, but wildly absurd theory lately introduced, first, if I mistake not, by the German poet

Goethe, a complete flower is represented to be the union of four whorls of leaves variously modified. In the same vein, Von Martius, of Munich, announces as a profound discovery that a plant is nothing but a leaf which has made a determinate number of revolutions, and hence all leaves ought by theory to grow alternately; but it being found that many leaves do not grow alternately, but one opposite to another, it is said this arises from the regular theoretical lengthening of the stem upwards being checked till the opposite leaf expands, a check that is uniform in the seed-leaves of those plants which have two seed-lobes. I submit to any reader endowed with common sense, that this is not science, but fanciful romance, of similar character to Van Helmont's Archæus and Darwin's gnomes and sylphs, though it is loudly trumpeted forth as being founded on rigid and accurate observation." (P. 43.)

However, he adopts Röper's views of the inflorescence founded upon it.

"Compound flower-stalks may be variously arranged and named, but I shall follow Professor Röper of Bâle, as the most modern, if not the best, in the midst of much confusion. Röper considers the modes of flowering as consisting of an evolution, which may be centripetal, centrifugal, or mixed. This arrangement, however, I ought to mention, evidently originated from the wild theory of Goethe and Von Martius, to which I have just objected; but as it is here introduced, I have completely stript it of all theory, and only given what is based on facts; for there is a wide difference between the representation of flowers being actually leaves transformed into flowers by rotatory evolution, and the simple fact that the flowers of certain plants are evolved in a certain order and direction." (P. 44.)

In attempting, however, to adopt this system, he flounders about as usual, calling the inflorescence of lavender a spike, when it is a thyrsus of verticillastri, and that of digitalis a spike, when it is a raceme.

At p. 114 he says correctly,

"Linnæus describes certain seeds as naked; but the envelope of the seed-organ is, with very few exceptions, such as in firs and pines, and the sago plant, never wanting, though sometimes it is so thin as not to be easily seen. It is always composed of an outer membrane, a middle membrane, and an inner membrane, all intimately united. These parts are very distinct in the peach, but not obviously distinct in the nut." (P. 114.)

But afterwards, to shew the reader that he did not understand the bit we have just quoted, our alphabet-monger confounds seeds and fruits in a most inexplicable manner; as thus,

"Seeds may be either close, or dehiscent. A close seed may be a grain, like wheat, maize, and rye-grass; it may be a simple pip, as in thistle and sunflower; it may be a composite pip, as in borage, dead nettle, lady's bed straw, ranunculus, parsley, and hemlock; it may be a key, as in ash, maple, and elm; it may be a gland, as in the oak and filbert; or it may be a utricle, as in the lime, the nettle, and orach.

"A dehiscent seed may be a follicle, as in the laurel rose; it may be a double follicle, as in swallow wort; it may be a purse, as in the cabbage and wallflower; it may be a purselet, as in honesty and shepherd's purse; it may be a pod, as in the pea, the bean, bladder senna, cassia, and astragalus; it may be a capsule, as in the pimpernel, poppy, and pinks; it may be a caper, as in spurge; or it may be a cone, as in alder, birch, and fir.

"*Fruits*, as popularly distinguished from seed, are more succulent or fleshy, and are all close. Botanists consider fruits to be the ripened seed-organ. A fruit may be a stone fruit, as the plum, the cherry, and the haw; it may be a nut, as the walnut and the almond; it may be a nutlet, as in the ivy; it may be a pome, as the apple, pear, and hip; it may be a pepon, as the melon and cucumber; and it may be a berry, as the vine, potato, gooseberry, orange, and fig; but the strawberry and raspberry rank as seeds with composite pips; and the mulberry and pine apple as a compound berry." (P. 125.)

Who ever heard of *seeds* being dehiscent and indehiscent? The grains of wheat, maize, and rye-grass, are not seeds, but fruits, *i. e.* seeds invested with their pericarps; and the same is true of every example given in the paragraph on close seeds. Then, who ever dreamed before of a follicle being a dehiscent seed; or of siliques and silicles, legumes and capsules, cocca and strobiles, (as in the wallflower, the pea, the bean, the poppy, and the fir,) being *seeds*, differing only in their dehiscence? Again, with regard to fruits, the examples are erroneous. A haw is not a drupe, but a pome; while the walnut and the almond, which the Professor calls nuts, are both drupes; the hip, which he calls a pome, is no such thing; the fruit of the spurge, which he terms an Elaterium, is as unlike one as possible; and as to botanists ranking, as he says they do, the strawberry and raspberry as seeds with composite pips, and the mulberry and pineapple as a compound berry, the very suggestion savours of madness. And yet this is the man who says "*I*" agree with Mirbel, or "*I*" differ from De Candolle, and who ventures, with the calm assurance of ignorance, to say what follows of Loudon's Encyclopædia of Plants, one of the most valuable and learned works that has been published for years: "Were I the proprietor of this work, I would not hesitate an instant to break up the stereotype plates, in order to expunge such glaring contradictions and highly dangerous errors." (P. 182.)

These specimens may serve to shew our younger readers in what the difficulty of compiling really consists. It does not consist in stringing together amusing bits from a number of books, for this our author can do; but in making them cohere, so as to form one harmonious whole, and this he cannot do. Moreover, the compiler must not only prevent new errors from gliding in, but must correct those which he finds; and this, again, our compiler cannot do. The man who has none of these substantial titles to praise, but rests his hopes on neat printing, green covers, and the applause of the newspapers, may be assured that the *Manchester Courier* and the *Bristol Mercury* are not the harbingers of scientific fame; that the critics (though few there be,) who understand the subject of their examination, are the real Court of Cassation, to whose decrees all inferior tribunals must submit; while the reputation which Professor Rennie is endeavouring to establish is as ephemeral as the periodicals which trumpet it forth.

